# Access for all: the Healthy Outlook^®^ service delivery model in Moray

**Published:** 2012-06-15

**Authors:** Lorna Gail Bernard

**Affiliations:** Moray Community Health and Social Care Partnership, UK

**Keywords:** COPD, health forecasting, self-care

## Abstract

Moray effectively changed the way in which the Met Office’s Healthy Outlook service was being delivered in order to make it more accessible and inclusive for GPs and more importantly, their patients with Chronic Obstructive Pulmonary Disease (COPD). The Met Office’s Healthy Outlook^®^ service supports people with COPD to take control of their own health care. The service represents a high impact, low resource Telehealth intervention which uses automated telephone prompts to alert people when their health is more likely to be compromised because of environmental conditions. The calls then prompt people to take simple precautions to manage their condition. The service was offered in Moray since 2007 but take-up was initially poor. This was believed to be because of the way in which the service is traditionally offered—typically commissioned and paid for by Health Authorities/Partnerships, then made available to GPs to offer to patients. This effectively made GPs ‘gatekeepers’ of the service, thus creating inequality in a ‘postcode lottery’ situation.

The service delivery model was therefore changed by trial in 2009/2010. Administration was centralised thus allowing patients to self-refer, thus empowering them to make their own choices about what might be beneficial to support them. Evaluation was extremely positive resulting in the service being adopted by the NHS locally as core business since January 10, 2011. Morays remain the pioneers of this service delivery model.

